# Supplementary material for: Risk factors associated with Shigella diarrhea in 6–35-month-olds: A cross-sectional study, Malawi, 2022–2024
Source: PLOS Glob Public Health. 2026 Apr 10;6(4):e0005374. doi: 10.1371/journal.pgph.0005374 (PMC13068323; doi:10.1371/journal.pgph.0005374)
Supplement: Attachment — Submitted filename: Responses to reviewers comments.pdf [file pgph.0005374.s001.pdf]

RESPONSES TO REVIEWER COMMENTS

Risk factors associated with *Shigella* diarrhea in 6-35-months-olds: A Cross-Section Study, Malawi, 2022-2024. (PGPH-D-25-03065)

Reviewer #1

This paper examines factors associated with Shigella-attributed diarrhea among children aged 6–35 months in Malawi, including a novel assessment of seasonal effect modification. The analyses are technically rigorous and appropriately applied to the observational dataset, and the findings provide valuable evidence to guide targeted interventions, including the forthcoming Shigella vaccine rollout. I recommend publication pending a few minor revisions noted below.

Major Comments for the Author:

Comment:

The introduction explicitly situates the burden of Shigella in an economic context, but are there any other lenses, perhaps more human-centric, through which we can think about the implications of this burden?

Response: Thank you for this insightful comment. We have revised the introduction (lines 50-54) to include a more human-centric perspective on the burden of *Shigella*. Specifically, we added: "In addition to acute morbidity, *Shigella* infection and other enteric pathogens have been associated with linear growth faltering and stunting in early childhood, as observed in large multicountry birth cohort and case control studies. Early childhood stunting is linked to long-term adverse outcomes, including impaired cognitive development, lower educational attainment, and diminished capacity to reach full developmental potential in adulthood". This revision situates the burden of *Shigella* not only in economic terms but also in terms of its lasting impact on child development and human potential.

Comment:

Please include references for the categorization methods of diarrhea severity and WASH in the "Predictor Variables" section of the Methods.

Response: Thank you. The methods section has been updated to include the requested citations. We have added a reference to the WHO/UNICEF Joint Monitoring Programme (JMP) 2018 core questions document for household WASH definitions, as well as references supporting the severity definitions used in the analysis, including the modified Vesikari score (MVS) and the Global Enteric Multicenter Study (GEMS).

Comment:
How did you approach producing age group bins for this study? Was it data driven or decided a priori based on some contextual motivation?

Response: Thank you for the question. Age group bins were selected a priori based on the differential distribution of *Shigella* burden among young children. It is well known that Shigella incidence peaks in the second year of life (Platts-Mills et al.), making it important to differentiate between infants younger than 12 months, children 12-23 months, and older children. We balanced these important distinctions with keeping age bands wide enough so that there were enough participants in each group to enable meaningful comparisons.

Comment:
Please add a statement justifying Poisson regression as the chosen analytic method.

Response: For cross-sectional data with common outcomes, using logistic regression can lead to inflated estimates of risk as odds ratios cannot be reliably interpreted as prevalence or risk ratios (Gnardellis et al.). Therefore, modified Poisson regression is a more suitable analytic approach for our data and research questions. We have added some context to the methods section – thank you for your thoughtful comment and opportunity to clarify our methodological approach.

Comment:
Given that some samples were tested by culture, some by qPCR, and some by both it would be beneficial to add more clarification in the methods about the different testing procedures and results classification. For the samples tested by both methods, what happened if one result was positive and the other negative? In the discussion you state "Notably, 43% of qPCR-positive

cases were also culture-positive, supporting the clinical relevance of the qPCR-detected cases", however only 43% overlap between the two methods actually seems quite low – can you point to any other studies that have looked at this?

Response: Thank you for this comment, we have clarified this point in the methods section of the manuscript under outcome variable (lines 110-113). All participants were tested for *Shigella* using both culture and qPCR methods. The primary outcome was *Shigella* detected by either culture or qPCR, and that in sensitivity analysis, we examined two versions of the outcome, defined as *Shigella* identified by culture and *Shigella* identified by qPCR, respectively and in the discussion section (lines 312-314). qPCR is a more sensitive method, meaning that even though there is only 43% "overlap" with culture, it is identifying many *Shigella* infections that culture may miss (Pavlinac PB et al.)

Comment:

I believe when using generalized estimating equations (GEE) to account for clustering it is standard to report the number of clusters and distribution of cluster size.

Response: Thank you for your suggestion. We have added this to the results section (lines 172-173).

Comment:

These analyses rely on an assumption of missing data at random/completely at random, however the complete case analysis conducted excludes 32% of children with missing vaccination data. Please elaborate on this missingness in the limitations beyond reduction in sample size to include the potential bias introduced if the data is not in fact missing randomly. Alternatively, it may be worthwhile to consider inclusion of the observations with unknown vaccination status as a third category – which may still have relevant interpretation given the reality of often not knowing children's vaccination status when designing interventions.

Response: We thank the reviewer for their thoughtful critique and have followed the suggested approach of incorporating children with “unknown” vaccination status as a separate category of the age-appropriate vaccination variable. We opted to use this approach to reduce potential bias from missingness and to maintain our a priori assumption that age-appropriate vaccination is a relevant characteristic that should be accounted for in multivariate models. Our results were similar after changing age-appropriate vaccination to incorporate unknown vaccination status, and we updated the text accordingly in the results (lines 183-201) and in the discussion (lines 296-304). We recognize that this approach may collapse a group with unobserved heterogeneity and have acknowledged this limitation in the discussion (lines 318-320).

Comment:
Was there any consideration of prior antibiotic use among patients reporting to the clinic with diarrhea? Please elaborate how this may or may not influence these results (perhaps in the limitations).

Response: Thank you for this important comment. We have added text to the discussion (lines 324-327noting that we chose not to examine prior antibiotic use in our analysis. This was due to the challenges of interpreting model results in the context of high documented rates of antimicrobial resistance in *Shigella*, as well as the potential unreliability of caregiver-reported antibiotic type prior to enrollment.

Comment:
Please standardize spelling of “enrollment” throughout the manuscript (sometimes one vs two ls).

Response: Thank you for noting this inconsistency. We have standardized the spelling of “enrollment” throughout the manuscript.

Comment:
In Table 1 the Wasting “None” group percentage needs a decimal instead of a comma.

Response: Many thanks for identifying this error. The percentage in the Wasting "None" group in Table 1 has been corrected to use a decimal instead of a comma.

Reviewer #2

The findings are interesting but it need through revision considering the following critical points.

Comment:

Clearly mention the inclusion and exclusion criteria for selection of patients in the current study?

Response: Thank you for this comment. The inclusion and exclusion criteria for participant selection are described on lines 94-98.

Comment:

What was the limitation of the study?

Response: Thank you for the question. The limitations of the study are discussed on lines 317-328 of the discussion section and have been further amended based on reviewer feedback.

Comment:

Shigella were isolated and identified using culturing. What was the specie distribution of Shigella?

Response: Thank you for this question. While this question is outside the scope of the current paper, the serotype distribution of *Shigella* cases in EFGH was reported in the following paper: (Yousafzai M T et al.)

Comment:

Mention the duration of the study (months/years) in the abstract.

Response: Many thanks for this suggestion. We have addressed this comment by adding the duration of the study to the Abstract (line 26).

Comment:
Briefly describe how the culture and qPCR were used for detection of Shigella. Is Shigella DNA directly detected in fecal sample or it is detected from culture?

Response: Thank you for this comment. All participants were tested for *Shigella* using both culture and qPCR methods independently. Both methods tested rectal swab samples. The primary outcome was defined as *Shigella* detected by either method, and sensitivity analyses examined culture-positive and qPCR-positive outcomes separately. We have updated text in the methods (lines 110-113) to clarify these definitions.

Comment:
Define the criteria of Household drinking water source categorization: Improved, Unimproved??

Response: Thank you for this comment. We have clarified the criteria used to categorize household drinking water sources as improved or unimproved in the Methods section under (lines 137-138) and have added a citation to the WHO/UNICEF Joint Monitoring Programme (JMP) 2018 report, as suggested.

Comment:
The abbreviations used in the tables should be defined in the table's foot note.

Response: Thank you for your attention to detail, we have added footnotes clarifying abbreviations to all tables.

References

Kotloff KL, Nataro JP, Blackwelder WC, Nasrin D, Farag TH, Panchalingam S, et al. Burden and aetiology of diarrhoeal disease in infants and young children in developing countries (the Global Enteric Multicenter Study, GEMS): a prospective, case-control study. Lancet. 2013 Apr;382(9888):209-222. doi:10.1016/S0140-6736(13)60844-2.

World Health Organization; United Nations Children's Fund; WHO/UNICEF Joint Monitoring Programme for Water Supply, Sanitation and Hygiene (JMP). Core questions on drinking water, sanitation and hygiene for household surveys: 2018 update. New York: United Nations Children's Fund (UNICEF) and World Health Organization; 2018. ISBN: 978-92-806-5009-9.

Gnardellis C, Notara V, Papadakaki M, Gialamas V, Chliaoutakis J. Overestimation of relative risk and prevalence ratio: misuse of logistic modeling. Diagnostics (Basel). 2022 Nov 17;12(11):2851. doi:10.3390/diagnostics12112851. PMID: 36428910; PMCID: PMC9689401.

Pavlinac PB, Platts-Mills JA, Tickell KD, Liu J, Juma J, Kabir F, Nkeze J, Okoi C, Operario DJ, Uddin J, Ahmed S, Alonso PL, Antonio M, Becker SM, Breiman RF, Faruque ASG, Fields B, Gratz J, Haque R, Hossain A, Hossain MJ, Jarju S, Qamar F, Iqbal NT, Kwambana B, Mandomando I, McMurry TL, Ochieng C, Ochieng JB, Ochieng M, Onyango C, Panchalingam S, Kalam A, Aziz F, Qureshi S, Ramamurthy T, Roberts JH, Saha D, Sow SO, Stroup SE, Sur D, Tamboura B, Taniuchi M, Tennant SM, Roose A, Toema D, Wu Y, Zaidi A, Nataro JP, Levine MM, Houpt ER, Kotloff KL. *The clinical presentation of culture-positive and culture-negative, quantitative polymerase chain reaction (qPCR)-attributable shigellosis in the Global Enteric Multicenter Study and derivation of a Shigella severity score: implications for pediatric Shigella vaccine trials.* Clin Infect Dis. 2021;73(3):e569–e579. doi:10.1093/cid/ciaa1545.

Yousafzai M T, Cornick J, Peñataro Yori P, Hossain M J, Keita A M, Atlas H E, Khanam F, Omore R, Galagan S R, Ahmed N, Ahmmed F, Awuor A O, Badji H, Conteh B, Garcia Quesada M, Garcia-Bardales P F, Horne B, Hotwani A, Houpt E R, Islam M T, Jere K C, Juma J, Liu J, Mategula D, Ogwel B, Okonji C, Onwuchekwa U U, Paredes Olortegui M, Platts-Mills J A, Qureshi S, Rajib N H, Rogawski McQuade E T, Schiaffino F, Secka O, Sow S O, Witte D, Kotloff K L, Cunliffe N A, Clemens J D, Tennant S M, Qamar F N, Kosek M N, Pavlinac P B, Tapia M D, Ochieng J B, EFGH Consortium. *Incidence and Antimicrobial Resistance of Shigella-Attributable Diarrhea in Young Children: Results from the Multi-Country Enterics for Global Health (EFGH) Shigella Surveillance Study.* SSRN Electron J [preprint]. 2025 Aug 12 [cited 2026 Feb 11]; Available from: https://ssrn.com/abstract=5386776

Platts-Mills JA, Liu J, Rogawski ET, Kabir F, Lertsethtakarn P, Siguas M, et al. Pathogen-specific burdens of community diarrhoea in developing countries: a multisite birth cohort study (MAL-ED). Lancet Glob Health. 2015;3(9):e564–e575. doi:10.1016/S2214-109X(15)00151-5.
